# Functional Characterization of *IbHK1a* Reveals Its Role in Enhancing Drought and Salt Tolerance Through Reactive Oxygen Species Regulation and Two-Component System Signaling in Sweet Potato (*Ipomoea batatas* L.)

**DOI:** 10.3390/plants15162507

**Published:** 2026-08-19

**Authors:** Ruxue Huo, Imran Khan, Jia Shi, Xuerui Li, Xiaoyu Cui, Shengjie Dai, Xiaohua Wang, Hongxia Zhang, Zongyun Li, Zhenning Liu

**Affiliations:** 1 College of Agriculture and Forestry Sciences, Linyi University, Linyi 276000, China; 2 School of Life Sciences, Jiangsu Normal University, Xuzhou 221116, China

**Keywords:** sweet potato, histidine kinase, *IbHK1a*, two-component system (TCS), drought stress, salt stress, reactive oxygen species, antioxidant enzymes

## Abstract

Drought and salinity are major abiotic stresses that severely constrain plant growth and agricultural productivity. Histidine kinases (HKs), as key components of the plant two-component system (TCS), play crucial roles in environmental signal perception and adaptive responses. In this study, we functionally characterized a sweet potato (*Ipomoea batatas* L.) HK gene, *IbHK1a*, and investigated its role in drought and salt stress tolerance. Expression analysis revealed that *IbHK1a* is predominantly expressed in root tissues, particularly in storage and fibrous roots, indicating its potential involvement in stress sensing and adaptation. Subcellular localization demonstrated that the IbHK1a protein is localized to the plasma membrane, suggesting a role in external signal perception. To elucidate its biological function, *IbHK1a* was heterologously overexpressed in *Arabidopsis thaliana*. Transgenic plants exhibited significantly enhanced tolerance to drought and salt stress, as evidenced by higher seed germination rates, improved primary root growth, reduced leaf wilting, and increased survival rates compared with wild-type (WT) plants. Physiological analyses showed that *IbHK1a* overexpression led to increased activities of antioxidant enzymes, including superoxide dismutase (SOD), peroxidase (POD), and catalase (CAT), accompanied by reduced accumulation of reactive oxygen species (ROS) such as hydrogen peroxide (H_2_O_2_) and malondialdehyde (MDA). Consistently, leaf histochemical staining confirmed lower ROS accumulation in transgenic plants under stress conditions. In sweet potato, overexpression of *IbHK1a* in transgenic hairy roots enhanced tolerance to drought and salinity, whereas RNA interference lines displayed increased sensitivity, further confirming its positive regulatory role. Additionally, protein interaction analysis indicated that *IbHK1a* interacts with *Arabidopsis* histidine phosphotransferase proteins (AHPs), suggesting its involvement in conserved TCS-mediated phosphorelay signaling pathways. Functional complementation analysis demonstrated that *IbHK1a* partially rescues the stress-sensitive phenotype of the *AHK1* mutant, indicating functional conservation with *Arabidopsis AHK1*. Collectively, these findings demonstrate that *IbHK1a* positively regulates drought and salt stress tolerance by enhancing antioxidant defense and ROS homeostasis. Its interaction with AHPs and partial complementation of the *ahk1* mutant further support its involvement in the conserved TCS phosphorelay pathway. These results establish *IbHK1a* as an important component of abiotic stress responses and a potential genetic target for improving drought and salinity tolerance in sweet potato.

## 1. Introduction

Drought and salinity are major abiotic stresses that adversely affect plant growth, development, and crop productivity worldwide [1,2,3]. These stresses disrupt cellular homeostasis, impair photosynthesis, and trigger excessive accumulation of ROS, leading to oxidative damage and yield reduction [4,5,6]. With increasing climate variability, enhancing plant tolerance to water deficit and salinity has become a major objective in crop improvement programs [7]. Therefore, elucidating the molecular mechanisms underlying plant stress perception and adaptive responses is essential. Plants have evolved sophisticated signaling networks to sense and respond to environmental cues, among which the TCS represents a key signaling pathway [8,9,10]. TCS proteins include HKs, histidine phosphotransfer proteins, and response regulators, forming a multistep phosphorelay system that transduces external stimuli into transcriptional responses [11,12]. In plants, TCS signaling is involved not only in cytokinin-mediated developmental regulation but also in responses to abiotic stresses such as drought, salinity, and cold [13,14,15,16]. HKs act as primary sensors in TCS signaling pathways, perceiving environmental signals and initiating downstream responses [10,11,17]. In *Arabidopsis thaliana*, several HKs have been functionally characterized, including *AHK1*, which positively regulates drought and salt stress tolerance [18], and *AHK5*, which integrates environmental and endogenous signals, including ROS and hormone signaling pathways [19,20]. These findings highlight the importance of HKs in abiotic stress adaptation; however, the functional roles and regulatory mechanisms of HK homologs in crop species remain largely unexplored.

Sweet potato (*Ipomoea batatas* L.) is an important root crop widely cultivated for its high nutritional value and adaptability to diverse environments [21,22,23]. It plays an important role in food security, particularly in regions prone to drought and salinity stress [24,25]. Despite its relatively strong tolerance, prolonged exposure to these stresses can severely impair growth, storage root development, and yield [26]. Although several stress-responsive genes have been identified in sweet potato, the molecular mechanisms underlying its stress tolerance, particularly the roles of TCS signaling in stress responses, warrant further investigation.

Recent studies have begun to identify TCS components in sweet potato, suggesting their involvement in abiotic stress responses. For example, HK genes have been reported to enhance drought and salt tolerance through modulation of ROS homeostasis and stress-responsive pathways. However, functional characterization of individual HK genes in sweet potato is still limited, and their roles in stress signaling require further investigation. In this study, we focus on *IbHK1a*, a sweet potato HK gene homologous to *Arabidopsis AHK1*. Expression analysis indicates that *IbHK1a* is predominantly expressed in root tissues, suggesting a role in environmental signal perception, given the established function of *AHK1* in abiotic stress responses [18]. In this study, we investigated the role of *IbHK1a*, a sweet potato HK homolog of *Arabidopsis AHK1*, in drought and salt stress responses. We hypothesized that *IbHK1a* functions as a positive regulator of abiotic stress tolerance. To test this hypothesis, we performed functional analyses using heterologous expression in *Arabidopsis thaliana* and transgenic hairy roots of sweet potato. Specifically, this study aimed to (i) characterize the expression pattern and subcellular localization of *IbHK1a*; (ii) determine its role in drought and salt stress tolerance; (iii) assess its effects on antioxidant defense and ROS homeostasis under stress conditions; and (iv) investigate its potential involvement in the conserved TCS phosphorelay pathway through protein interaction and functional complementation analyses. These findings provide insights into the function of *IbHK1a* in abiotic stress responses and its potential utility for improving stress tolerance in sweet potato.

## 2. Results

### 2.1. Tissue-Specific Promoter Activity Analysis of IbHK1a in Transgenic Arabidopsis thaliana

**Figure 1:**
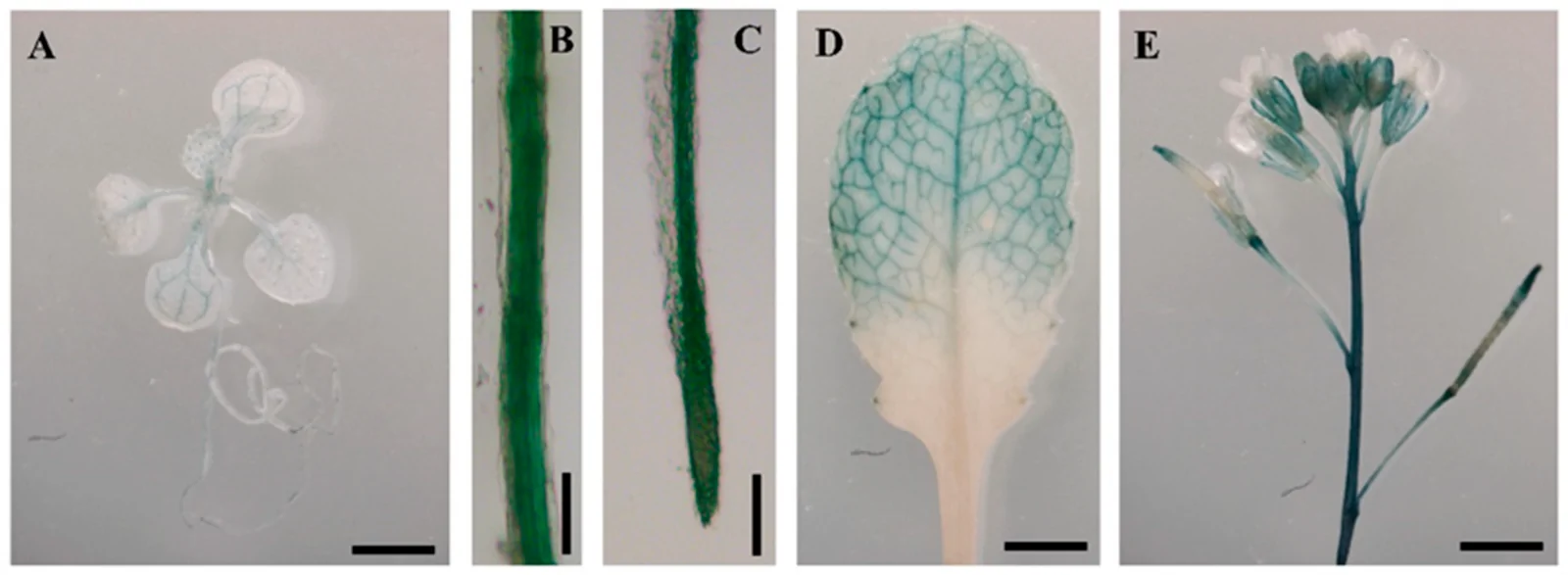
Histochemical GUS staining analysis of the *IbHK1a* promoter activity in transgenic *Arabidopsis thaliana*. GUS expression was detected in different tissues, including (**A**) seedling, (**B**) root, (**C**) root tip, (**D**) leaf, and (**E**) inflorescence. Scale bars =5 mm in all panels.

To investigate the spatial activity of *IbHK1a*, a promoter–reporter construct (*IbHK1apro::H2B-eGFP-GUS-NOST-pCAMBIA1300*) was generated and introduced into *Arabidopsis thaliana*. A total of 20 independent positive transgenic lines were obtained, and GUS staining was performed at different developmental stages and in various tissues. As shown in Figure 1A–E, GUS activity was detected in different tissues of transgenic plants. In 10-day-old seedlings, GUS staining was observed in both aerial and root tissues (Figure 1A). Strong GUS activity was detected along the root tissues (Figure 1B), while a more detailed examination revealed prominent GUS staining in the root tip and adjacent root regions (Figure 1C). These complementary observations indicate spatial activity of the *IbHK1a* promoter in different regions of the root. Strong GUS signals were also observed in the vascular tissues of rosette leaves, predominantly along the leaf veins (Figure 1D). In addition, clear GUS staining was detected in floral stems and floral organs (Figure 1E). GUS staining was also observed in siliques, where it was mainly concentrated at the apical region and at the junction between the silique and pedicel.

### 2.2. Localization of IbHK1a Protein in the Cell Membrane

**Figure 2:**
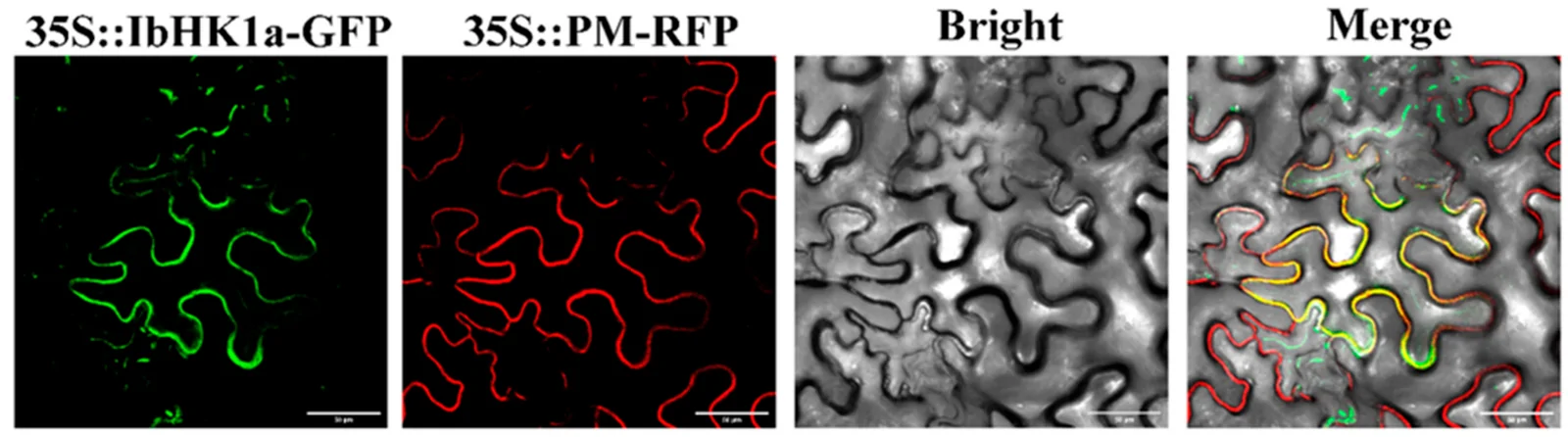
Subcellular localization of IbHK1a. Tobacco leaf epidermal cells were cotransformed with the fusion construct (IbHK1a-GFP) and the plasma membrane marker PIP2-mCherry, Scale bar = 50 μm.

To determine the subcellular localization of the *IbHK1a* protein, a fusion construct (35Spro::*IbHK1a*-eGFP-NOST-pCAMBIA1300) was generated and transiently expressed in *Nicotiana benthamiana* leaves. A plasma membrane marker (35Spro::PIP2-mCherry) was co-expressed as a reference. Using confocal laser scanning microscopy, fluorescence signals were observed. As shown in Figure 2, the *IbHK1a-eGFP* fusion protein exhibited green fluorescence predominantly at the cell membrane, which overlapped with the red fluorescence signal of the membrane marker. These results indicate that *IbHK1a* is localized to the plasma membrane.

### 2.3. Screening and Identification of Transgenic Arabidopsis Lines Overexpressing IbHK1a from Sweet Potato

To investigate the functional role of *IbHK1a*, an overexpression construct (*35Spro::IbHK1a-NOST-pCAMBIA1300*) was introduced into *Arabidopsis thaliana*. A total of 18 positive transgenic lines were obtained and confirmed by genomic PCR. Homozygous T3 lines were subsequently generated through selfing. The expression levels of *IbHK1a* in the transgenic lines were analyzed by qRT-PCR (Figure S1). The results showed variable expression among different lines, with L8, L13, and L17 exhibiting relatively higher expression levels, while no expression was detected in WT plants. These homozygous lines (L8, L13, and L17) were selected for subsequent analyses.

### 2.4. The Overexpression of the IbHK1a Gene Enhances the Drought and Salt Stress Tolerance in Arabidopsis

**Figure 3:**
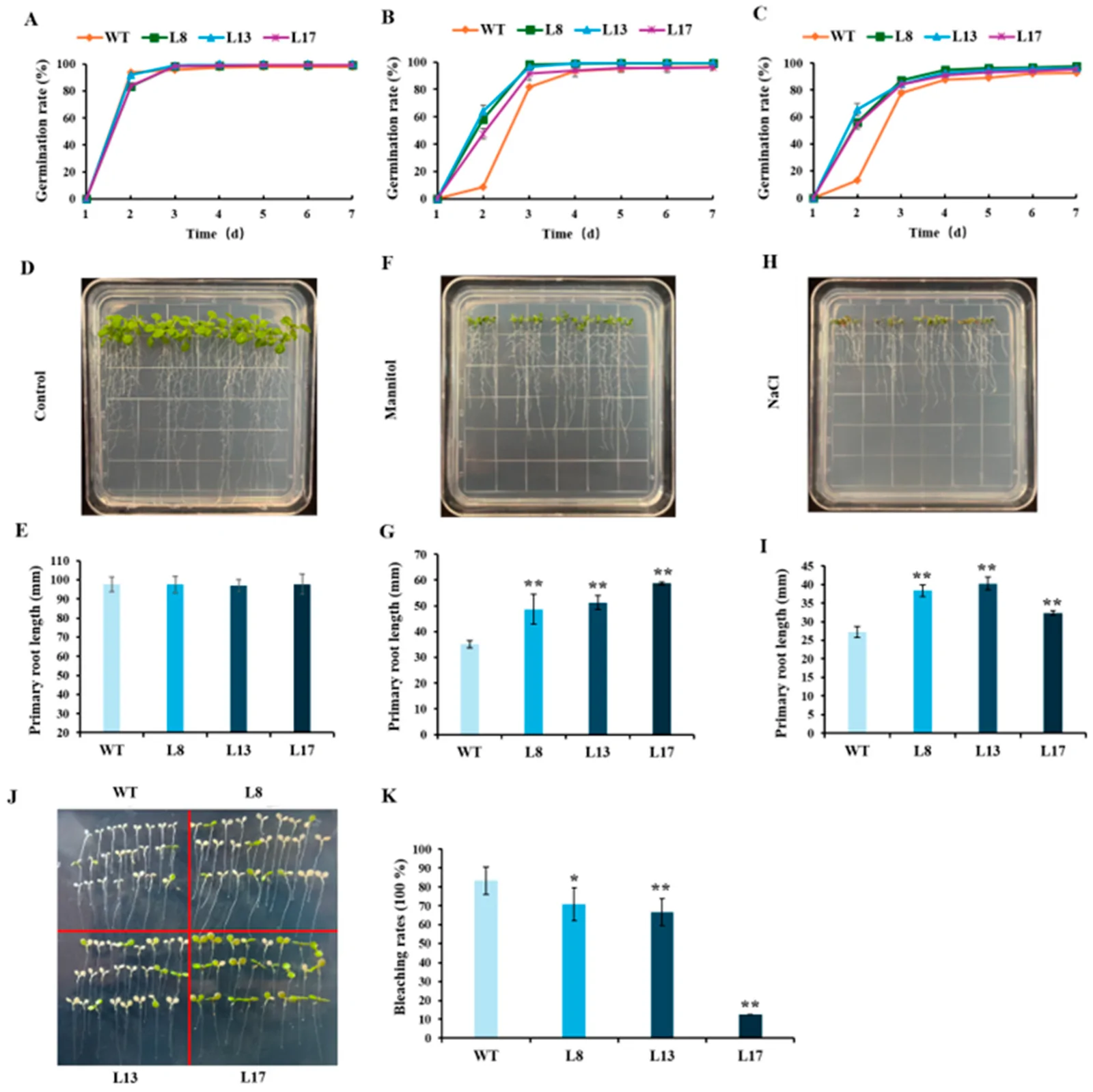
Germination assay of *IbHK1a*-OE transgenic *Arabidopsis* and WT seeds on MS medium. (**A**) control, (**B**) with 100 mM mannitol, and (**C**) with 100 mM NaCl, respectively. Seed germination was calculated at the indicated times (1, 2, 3, 4, 5, 6, 7 d). (**D**,**E**) Phenotypes and root length of WT and *IbHK1a*-OE lines (L8, L13, L17) grown for 2 weeks on MS medium under control conditions. (**F**,**G**) Root growth and quantitative analysis under osmotic stress (200 mM mannitol). (**H**,**I**) Root growth and quantitative analysis under salt stress (150 mM NaCl). (**J**,**K**) Salt tolerance assay of seedlings exposed to 250 mM NaCl for 5 days after initial growth on MS medium; representative phenotypes and chlorosis rates are shown. Data were collected from three biological replicates (*n* = 5 for root length; *n* = 24 for salt tolerance test). Student’s *t*-test was used to determine the statistical significance (* *p* < 0.05; ** *p* < 0.01).

To evaluate the effects of *IbHK1a* overexpression on stress tolerance during germination, seeds from homozygous T_3_ transgenic lines (L8, L13, and L17) and WT plants were subjected to germination assays under control, drought (100 mM mannitol), and salt (100 mM NaCl) conditions. Under control conditions, no significant differences in germination rates were observed among all lines, with >95% germination by day 3 and nearly 100% by day 7 (Figure 3A). Under mannitol treatment, transgenic lines exhibited markedly enhanced germination compared with WT. By day 2, germination rates of L8, L13, and L17 reached 58.3%, 64.0%, and 47.6%, respectively, whereas WT showed only 8.7%. By day 3, transgenic lines achieved >90% germination, significantly higher than WT (81.5%). By day 7, germination rates of all lines approached saturation, with transgenic lines remaining slightly higher than WT (Figure 3B).

Similarly, under salt stress, transgenic lines displayed accelerated and higher germination rates. On day 2, L8, L13, and L17 showed germination rates of 56.2%, 65.3%, and 54.8%, respectively, compared with 13.2% in WT. By day 3, transgenic lines maintained significantly higher germination than WT, and by day 7, all lines reached high germination levels, with transgenic lines consistently outperforming WT (Figure 3C). These results demonstrate that *IbHK1a* overexpression significantly enhances seed germination under both drought and salt stress conditions.

To further assess the role of *IbHK1a* in stress tolerance, 3- to 4-day-old seedlings with uniform growth were transferred to MS medium supplemented with 0, 200 mM mannitol, or 150 mM NaCl, and primary root length was measured after 10 days. Under control conditions, no significant differences in root length were observed among WT and transgenic lines (L8, L13, and L17), with all lines showing comparable growth (Figure 3D,E). Under drought stress (200 mM mannitol), transgenic lines exhibited significantly longer primary roots than WT. The average root lengths of L8, L13, and L17 were markedly higher than those of WT, indicating enhanced tolerance to osmotic stress (Figure 3F,G). Similarly, under salt stress (150 mM NaCl), transgenic lines maintained significantly greater root growth compared with WT, further supporting improved stress tolerance (Figure 3H,I). To evaluate salt-induced damage, 5-day-old seedlings were exposed to 250 mM NaCl for 5 days. WT plants showed severe chlorosis (83.3%), whereas transgenic lines displayed significantly reduced chlorosis, particularly L17, which exhibited minimal symptoms (Figure 3J,K). These results demonstrate that *IbHK1a* overexpression promotes root growth and reduces stress-induced damage under both drought and salt conditions.

**Figure 4:**
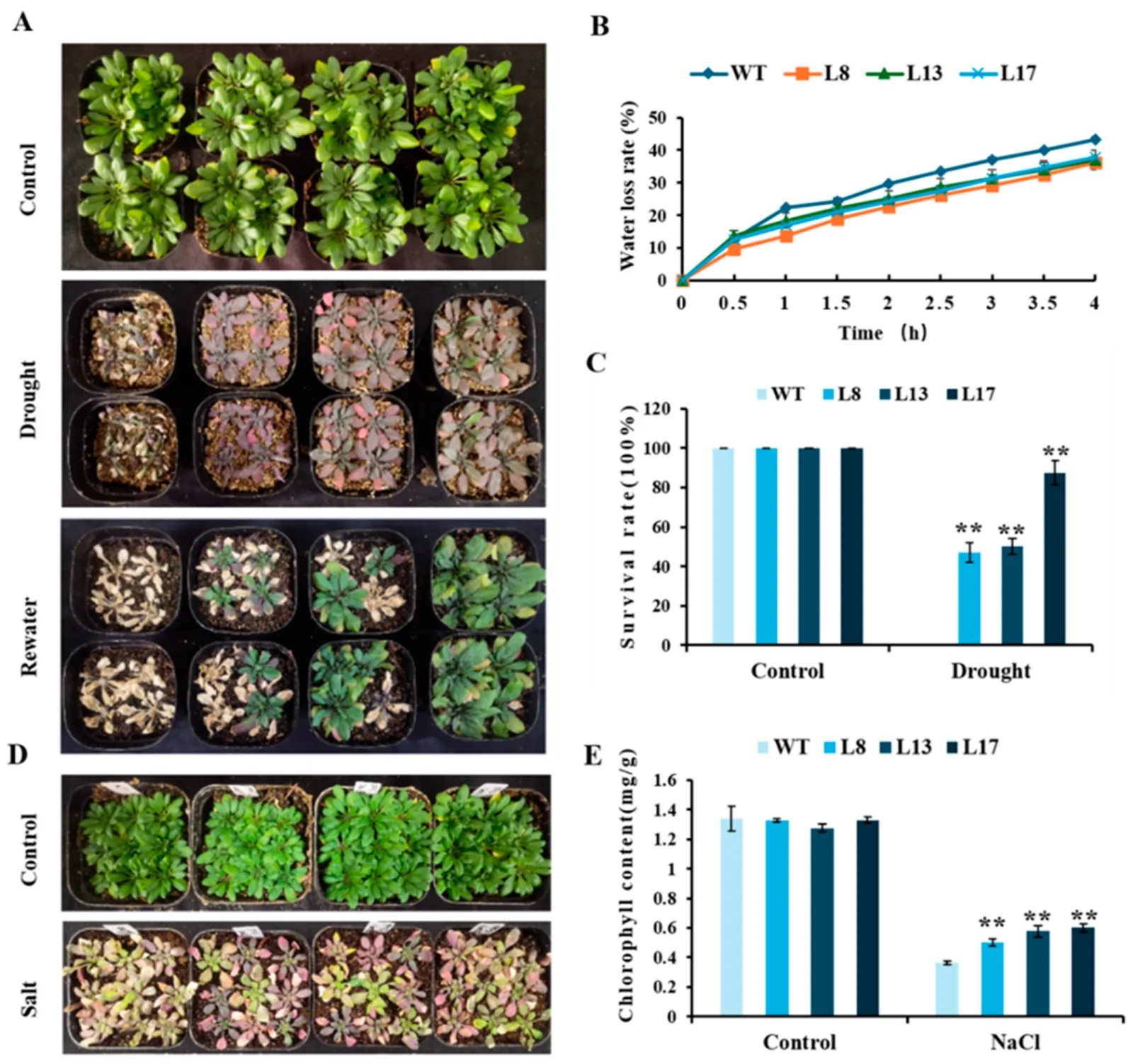
Responses of *IbHK1a*-overexpressing (OE) *Arabidopsis* lines and WT (WT) plants to drought and salt stress. (**A**) Phenotypic comparison of WT and transgenic plants under drought stress. Plants were first grown under normal conditions for 2 weeks, followed by 4 weeks of drought treatment, and subsequently rewatered for 3 days. Plants grown continuously under normal conditions for 6 weeks were used as the control. (**B**) Water loss rates of detached leaves collected from 4-week-old WT and transgenic plants under standard growth conditions were used to evaluate leaf dehydration tolerance. (**C**) Survival rates of WT and transgenic plants were assessed after 3 days of recovery following drought stress and rewatering. (**D**) Morphological phenotypes of WT and transgenic plants exposed to salt stress after 2 weeks of normal growth. Plants maintained under standard conditions for 6 weeks served as the control group. (**E**) Measurement of total chlorophyll content under different treatment conditions, reflecting the physiological status of the plants under stress. All data are presented as means from three independent biological replicates. Statistical significance was determined using Student’s *t*-test, where asterisks indicate significant differences (** *p* < 0.01).

To further validate the role of *IbHK1a* in abiotic stress tolerance, 2-week-old *Arabidopsis* seedlings were transplanted to soil and subjected to natural drought and salt treatments. Under normal conditions, no visible phenotypic differences were observed between WT and transgenic lines (L8, L13, and L17) (Figure 4A). However, detached leaves from transgenic plants exhibited a significantly lower water loss rate compared with WT (Figure 4B). Following approximately 4 weeks of drought stress, WT plants displayed severe wilting and desiccation, whereas transgenic lines showed only mild symptoms (Figure 4A). After rewatering for 3 days, transgenic lines exhibited markedly higher survival rates (46.9–87.5%) compared with WT, which failed to recover (Figure 4C). Under salt stress, WT plants exhibited more pronounced leaf yellowing than transgenic lines (Figure 4D). Consistently, chlorophyll content decreased in all plants under salt treatment, but was significantly better maintained in transgenic lines than in WT (Figure 4E). These results indicate that *IbHK1a* overexpression contributes to both drought and salt tolerance in *Arabidopsis* by reducing water loss and alleviating stress-induced damage.

### 2.5. The Overexpression of the IbHK1a Gene Enhances Antioxidant Enzyme Activity and ROS Scavenging Capacity in Arabidopsis

**Figure 5:**
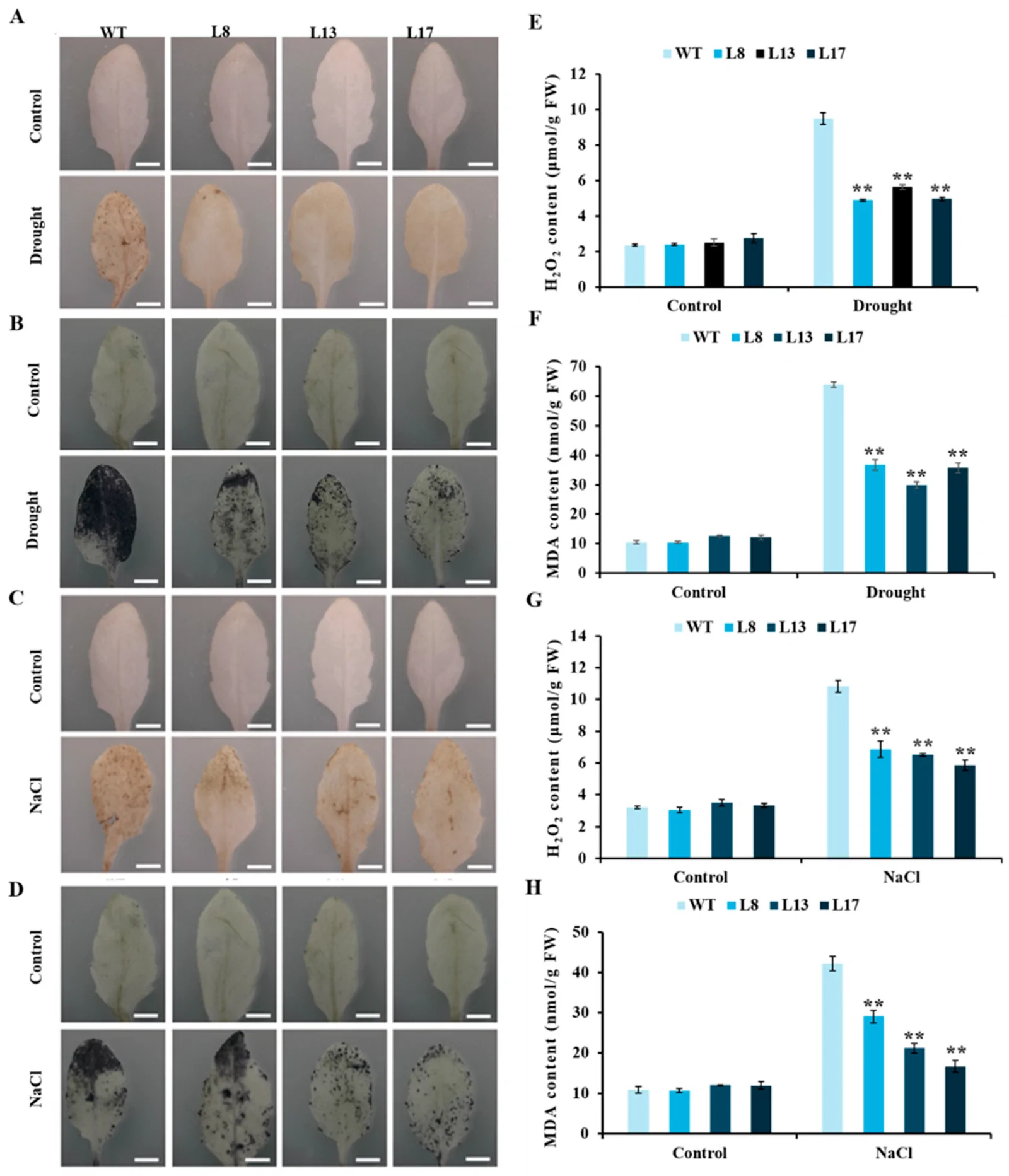
Detection of ROS in leaves of *IbHK1a* OE *Arabidopsis* lines and WT plants under drought and salt stress. (**A**,**B**) DAB staining and NBT staining under drought stress, (**C**,**D**) DAB and NBT staining under NaCl stress, (**E**,**F**) H_2_O_2_ and MDA content under drought stress, (**G**,**H**) H_2_O_2_ and MDA content under NaCl stress, respectively. Each treatment has three biological replicates (*n* = 3). ** Indicates *p* < 0.01, according to Student’s *t*-test. Scale bars =5 mm in all panels.

To further characterize the physiological responses associated with enhanced stress tolerance, ROS accumulation was examined in rosette leaves following 2 weeks of drought and salt stress. DAB and NBT staining were performed to detect the accumulation of H_2_O_2_ and O_2_^−^, respectively. Under control growth conditions, no obvious differences in DAB or NBT staining intensity were observed between WT and transgenic lines (L8, L13, and L17), indicating low levels of ROS accumulation in all plants (Figure 5A–D). However, after 2 weeks of drought and salt stress, WT leaves exhibited markedly stronger staining compared to transgenic lines, indicating higher accumulation of H_2_O_2_ and O_2_^−^ in WT plants (Figure 5A–D). Consistent with the staining results, quantitative measurements showed that WT plants accumulated significantly higher H_2_O_2_ levels and exhibited higher MDA contents than the transgenic lines under both drought and NaCl stress conditions (Figure 5E–H). In contrast, L8, L13, and L17 maintained significantly lower H_2_O_2_ levels and MDA contents under both stress conditions, indicating reduced ROS accumulation and lipid peroxidation in these transgenic lines.

**Figure 6:**
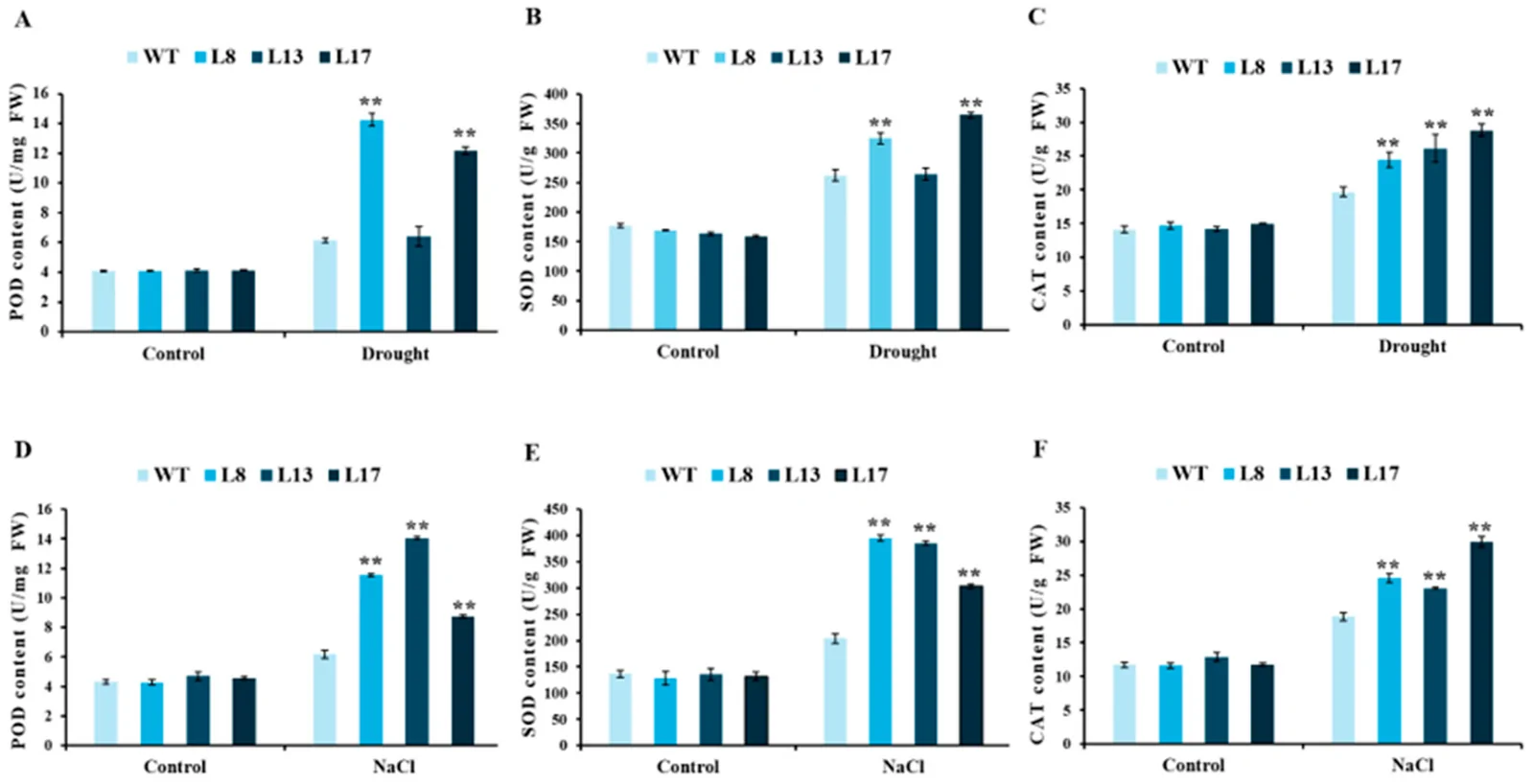
Determination of POD, SOD, and CAT activity in the leaves of the *IbHK1a*-OE transgenic and WT plants under drought and salt stress. (**A**) POD activity under control and drought conditions; (**B**) SOD activity under control and drought conditions; (**C**) CAT activity under control and drought conditions; (**D**) POD activity under control and NaCl treatment; (**E**) SOD activity under control and NaCl treatment; (**F**) CAT activity under control and NaCl treatment. Each treatment has three biological replicates (*n* = 3). ** Indicates *p* < 0.01, according to Student’s *t*-test.

The activities of key antioxidant enzymes, including POD, SOD, and CAT, were further analyzed. Under control conditions, enzyme activities were low and showed no significant differences among all lines (Figure 6). Following drought and salt stress treatments, the activities of POD, SOD, and CAT increased in all plants, respectively (Figure 6A–F); however, transgenic lines exhibited significantly higher enzyme activities compared to WT. These results indicate that overexpression of *IbHK1a* enhances ROS scavenging capacity and reduces oxidative damage under drought and salt stress conditions.

**Figure 7:**
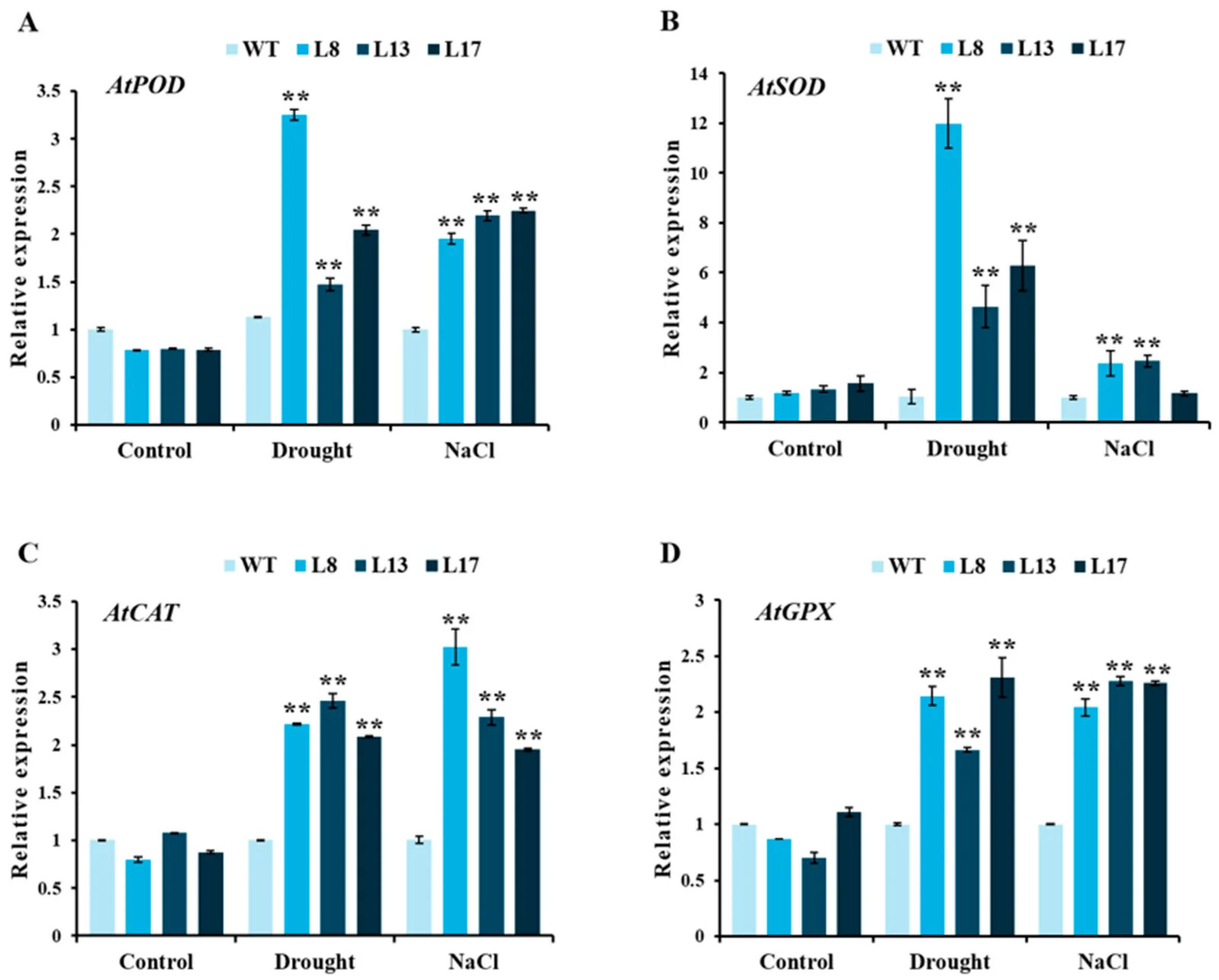
Expression levels of ROS-scavenging genes in the leaves of transgenic and WT plants under drought and salt stress. (**A**) Relative expression level of *AtPOD* under control, drought, and NaCl treatments; (**B**) relative expression level of *AtSOD* under control, drought, and NaCl treatments; (**C**) relative expression level of *AtCAT* under control, drought, and NaCl treatments; (**D**) relative expression level of *AtGPX* under control, drought, and NaCl treatments. Each treatment has three biological replicates (*n* = 3). ** Indicates *p* < 0.01, according to Student’s *t*-test.

In addition, the rosette leaves of *Arabidopsis thaliana* after 2 weeks of drought stress treatment and 2 weeks of salt treatment were selected to detect the relative expression levels of ROS scavenging system genes such as POD, SOD, CAT, and GPX by qRT-PCR, as shown in Figure 7. Under drought stress and salt stress treatment, the four genes *AtPOD*, *AtSOD*, *AtCAT*, and *AtGPX* in the *IbHK1a* overexpression lines showed relatively high expression levels compared with WT (Figure 7A–D, which was consistent with the higher POD, SOD, and CAT enzyme activity results in the *IbHK1a* overexpression lines, indicating that the overexpression of *IbHK1a* could activate the expression of genes related to the ROS scavenging system under drought stress and salt stress. This, in turn, improves antioxidant enzyme activity and ROS scavenging ability.

### 2.6. Genetic Transformation of Sweet Potato Adventitious Roots and Transgenic Line Screening

Research has demonstrated that heterologous overexpression of *IbHK1a* in *Arabidopsis thaliana* enhances drought and salt stress tolerance. However, its functional role in sweet potato remained unclear. To investigate the biological function of *IbHK1a* in sweet potato under abiotic stress conditions, an *Agrobacterium*-mediated adventitious root transformation system was employed to introduce the *pTNRT*::*IbHK1a* overexpression vector, *pTNRT:*:*IbHK1a* RNAi construct, and empty vector control (pTNRT) into sweet potato stem segments. The transformation system was optimized based on our established protocol. *Agrobacterium* suspensions were injected into internodal stem segments using a syringe, avoiding nodal regions. After injection, explants were cultured under normal growth conditions.

To validate transgenic status, fluorescence-positive roots were further confirmed by PCR and qRT-PCR. Genomic DNA was extracted from selected adventitious roots, and PCR amplification using vector-specific and *IbHK1a*-specific primers confirmed the presence of target fragments in fluorescence-positive lines (Figure S2). Expression analysis further demonstrated that *IbHK1a* transcript levels were significantly higher in pTNRT::*IbHK1a* overexpression lines and significantly reduced in pTNRT::*IbHK1a* RNAi lines compared with the empty vector control (pTNRT) Figure S2D. These results confirm the successful establishment of a sweet potato genetic transformation system and demonstrate effective modulation of *IbHK1a* expression in transgenic adventitious roots.

### 2.7. Positive Regulation of the IbHK1a Gene on Sweet Potato’s Tolerance to Drought and Salt Stress

**Figure 8:**
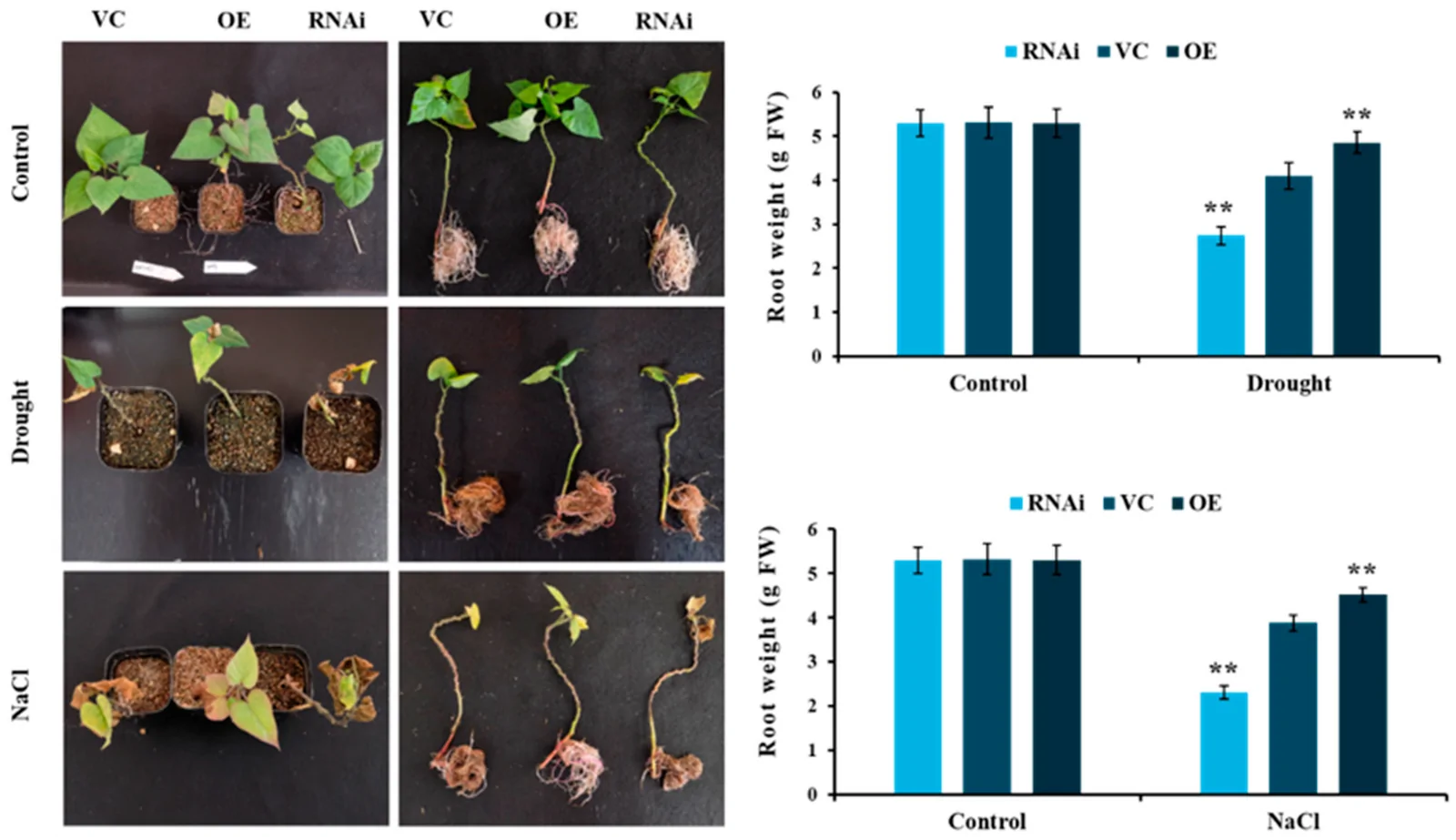
Phenotypes of *IbHK1a*-OE, *IbHK1a*-RNAi, and vector control (VC) sweet potato plants under normal conditions (control) or subjected to 20% PEG6000 and 300 mM NaCl, respectively. Representative photographs were taken after about 4 weeks of stress treatment. Each treatment had three biological replicates (*n* = 3). ** Indicates *p* < 0.01, according to Student’s *t*-test.

Healthy and uniformly growing transgenic sweet potato lines (*IbHK1a*-OE, *IbHK1a*-RNAi, and empty-vector control lines) were treated with Hoagland’s solution containing 20% PEG6000 or 300 mM sodium chloride (NaCl) to simulate drought and salt stress, respectively. Plants treated with normal Hoagland’s solution served as controls. After approximately 4 weeks of treatment, as shown in Figure 8, no significant phenotypic differences were observed in the leaves and adventitious roots of the *IbHK1a*-OE, *IbHK1a*-RNAi, and control lines in the control group. However, in the drought and salt stress treatment groups, compared to the control lines, the *IbHK1a*-OE lines showed better growth with less yellowing of the adventitious roots and greater fresh weight. In contrast, the *IbHK1a*-RNAi lines showed more severe leaf wilting, greater yellowing of adventitious roots, and smaller fresh weight, with significant differences. These results indicate that the overexpression of *IbHK1a* enhances sweet potato seedling tolerance to both drought and salt stress, while downregulation of *IbHK1a* expression increases sweet potato seedling sensitivity to these stresses.

### 2.8. Overexpression of IbHK1a Gene Increases Sweet Potato Antioxidant Enzyme Activity and ROS Clearance Capacity

**Figure 9:**
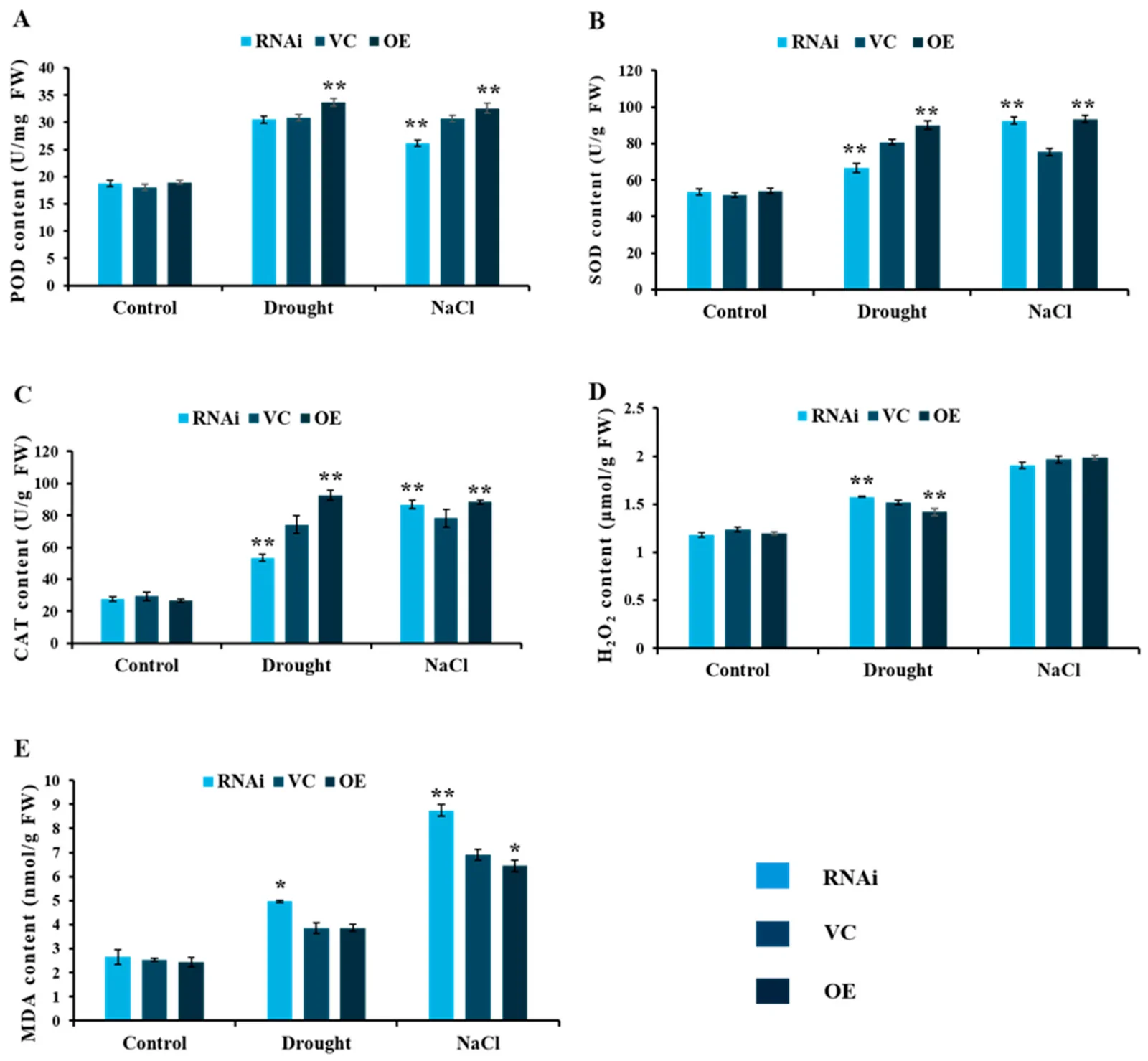
Determination of POD (**A**), SOD (**B**), and CAT (**C**) activity, and H_2_O_2_ (**D**), MDA (**E**) content in *IbHK1a*-OE, *IbHK1a*-RNAi, and vector control (VC) sweet potato hairy roots under normal and drought conditions, respectively. Each treatment has three biological replicates (*n* = 3). ** Indicates *p* < 0.01 and * Indicates *p* < 0.05 according to Student’s *t*-test.

The heterologous overexpression of *IbHK1a* in *Arabidopsis thaliana* enhances antioxidant enzyme activity and ROS scavenging capacity, thereby improving tolerance to drought and salt stress. To determine whether similar physiological mechanisms operate in sweet potato, antioxidant enzyme activities and oxidative stress indicators were measured in adventitious roots of transgenic sweet potato lines under drought and salt stress treatments. As shown in Figure 9, under stress conditions, *IbHK1a*-OE lines exhibited significantly higher activities of POD, SOD, and CAT compared with the empty vector control. In contrast, *IbHK1a*-RNAi lines showed reduced activities of these antioxidant enzymes (Figure 9A–C). Consistently, H_2_O_2_ and MDA contents were lower in *IbHK1a*-OE lines, while higher levels were detected in *IbHK1a*-RNAi lines (Figure 9D,E). These results indicate that *IbHK1a* enhances antioxidant enzyme activity and ROS scavenging capacity in sweet potato, thereby contributing to improved drought and salt stress tolerance.

### 2.9. Functional Conservation Between Sweet Potato IbHK1a and Arabidopsis AtAHK1 Homologous Genes

**Figure 10:**
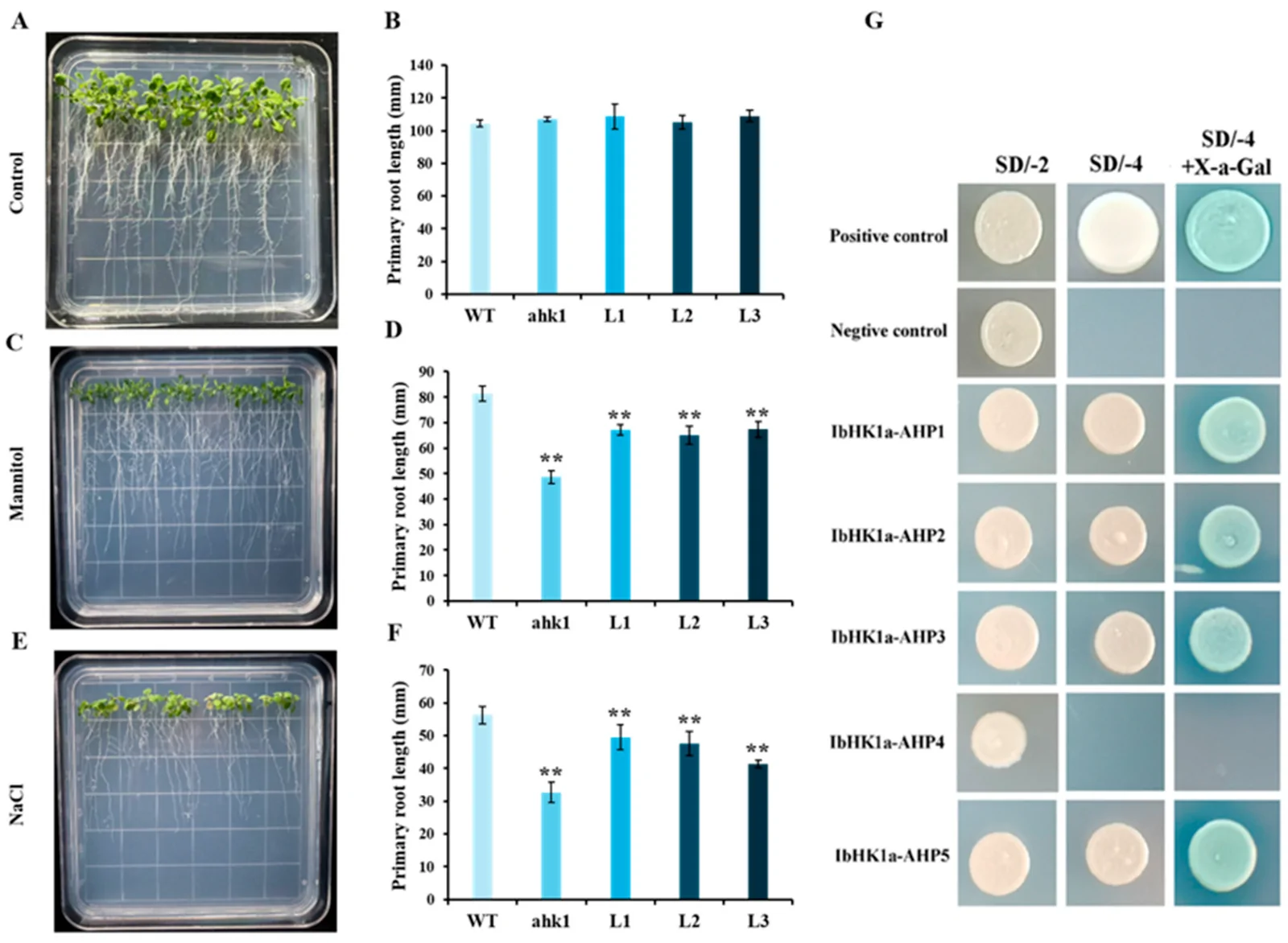
*IbHK1a* could partially rescue the *Arabidopsis AHK1* phenotype. Primary root growth of transgenic *Arabidopsis*, *ahk1* mutant, and WT seedlings cultured for 2 weeks on MS medium under different treatments. (**A**,**B**) Representative seedling phenotype and quantitative analysis of primary root length under control conditions; (**C**,**D**) representative seedling phenotype and quantitative analysis under 200 mM mannitol treatment; (**E**,**F**) representative seedling phenotype and quantitative analysis under 150 mM NaCl treatment. (**G**) Yeast two-hybrid analysis of protein–protein interactions between IbHK1a and Arabidopsis AHP proteins. Root-growth assays were performed with five seedlings per line in each of three independent biological replicates (*n* = 5 seedlings per biological replicate). ** Indicates *p* < 0.01, according to Student’s *t*-test.

Previous studies have reported that *Arabidopsis thaliana AHK1* positively regulates drought and salt stress responses, and the *AHK1* mutant exhibits increased sensitivity to both stresses. In the present study, the sweet potato homolog *IbHK1a* was also shown to positively regulate drought and salt stress tolerance. To investigate functional conservation between *IbHK1a* and *AtAHK1*, a complementation construct (*AHK1pro::IbHK1a-NOST-pCAMBIA1300*) was generated to drive expression of *IbHK1a* genomic DNA under the *AHK1* promoter and introduced into the homozygous *AHK1* mutant background. Homozygous complemented lines (*AHK1*/*AHK1*pro::*IbHK1a*) were obtained through screening. Seedlings of complemented lines, *AHK1* mutants, and WT plants were transferred to MS medium supplemented with 0, 200 mM mannitol, or 150 mM NaCl. The primary root length was measured after two weeks of growth. Control conditions revealed no significant differences among WT, *AHK1*, and complemented lines (Figure 10A,B). Under 200 mM mannitol treatment, WT seedlings exhibited an average root length of 81.3 mm, whereas *AHK1* mutants showed a significantly reduced root length of 48.7 mm. The complemented lines showed partial recovery, with root lengths of 67.2 mm, 65.0 mm, and 67.3 mm, respectively Figure 10C,D). A similar trend was observed under 150 mM NaCl treatment, where WT and *AHK1* exhibited average root lengths of 56.25 mm and 32.7 mm, respectively, while complemented lines showed intermediate values of 49.5 mm, 47.5 mm, and 41.3 mm (Figure 10E,F). These results indicate that *IbHK1a* partially rescues the drought- and salt-sensitive phenotype of the *AHK1* mutant, suggesting functional conservation between *IbHK1a* and At*AHK1* in stress responses.

To further investigate the molecular basis of this functional conservation, yeast two-hybrid assays were performed to examine interactions between IbHK1a and *Arabidopsis* histidine phosphotransfer proteins (AHP1–AHP5). The results showed that IbHK1a interacts with AHP1, AHP2, AHP3, and AHP5 (Figure 10G). These findings suggest that heterologously expressed *IbHK1a* can participate in the endogenous two-component signaling pathway in *Arabidopsis* by interacting with native AHP proteins, thereby mediating downstream signal transduction.

## 3. Discussion

Histidine kinases (HKs) represent critical sensory components in plant signal transduction, enabling plants to perceive and respond to environmental fluctuations through multistep phosphorelay systems [27,28]. In this study, functional characterization of the sweet potato HK gene *IbHK1a* demonstrates its essential role in enhancing tolerance to drought and salt stress, highlighting its importance as a regulator of stress adaptation in crop species. Plant adaptation to drought and salinity requires coordinated regulation of stress perception, signal transduction, cellular homeostasis, and downstream physiological responses. Recent studies have emphasized the importance of integrated regulatory mechanisms in crop drought resistance [29], while manipulation of stress-responsive regulatory genes, such as ZmASR6 in maize, can improve salt tolerance through modulation of cellular protective mechanisms [30]. In this context, the positive effects of *IbHK1a* under both drought and salinity suggest that this HK may represent an important regulatory component connecting stress-associated signaling with downstream adaptive responses.

The preferential expression of *IbHK1a* in root tissues (Figure 1) suggests a role in sensing soil-derived environmental signals, such as water deficit and ionic stress. Roots serve as the primary interface between plants and their environment, and root-specific expression of stress-responsive genes is often associated with improved adaptation to abiotic stress [31,32,33]. Because roots directly encounter changes in soil water availability and ion concentrations, they play a central role in the early perception of drought and salinity and in initiating subsequent adaptive responses [34]. Stress-induced modifications of root growth and architecture can further contribute to adaptation by influencing water and nutrient acquisition under unfavorable soil conditions [35]. Therefore, the preferential accumulation of *IbHK1a* transcripts in roots may facilitate an early response to changes in the rhizosphere and contribute to subsequent whole-plant stress acclimation. Moreover, the localization of *IbHK1a* to the plasma membrane (Figure 2) is consistent with its proposed function as a sensor protein, as membrane-localized HKs are known to detect extracellular signals and initiate downstream signaling cascades [11,36]. This localization pattern aligns with previous studies showing that membrane-associated kinases play pivotal roles in abiotic stress perception and signal initiation [37,38]. Together, the root-preferential expression and plasma membrane localization of *IbHK1a* are consistent with its proposed involvement in an early stage of stress-associated signaling. However, plasma membrane localization alone does not establish that *IbHK1a* directly perceives drought- or salt-derived signals, and its specific upstream stimulus remains to be determined.

The enhanced stress tolerance observed in *IbHK1a*-overexpressing *Arabidopsis* plants further supports its functional role in stress adaptation. Improved seed germination under osmotic and salt stress conditions (Figure 3A–C) indicates that *IbHK1a* mitigates early-stage stress inhibition, a critical determinant of plant establishment under adverse environments [39]. Seed germination and early seedling establishment are particularly sensitive to salinity because osmotic limitation and ion-specific effects interfere with water uptake and early metabolic processes [40]. Therefore, the higher germination rate of *IbHK1a*-overexpressing plants suggests that the protective function of *IbHK1a* is already evident during early plant development. Additionally, increased primary root growth under stress (Figure 3) suggests that *IbHK1a* contributes to maintaining root system development, which is essential for water and nutrient acquisition during stress conditions [41]. Enhanced root elongation has been widely associated with improved drought tolerance, as it allows plants to access deeper water reserves [31].

The reduction in seedling bleaching under salt stress (Figure 3J,K) further indicates that *IbHK1a* enhances cellular stability under ionic stress conditions. Salt stress disrupts ion homeostasis and causes chlorophyll degradation, leading to reduced photosynthetic efficiency [42,43]. The improved chlorophyll retention observed in transgenic plants (Figure 4) suggests that *IbHK1a* helps maintain photosynthetic capacity under stress, a trait strongly associated with stress tolerance and yield stability [44,45]. Similarly, reduced water loss and increased survival rates under drought stress (Figure 4) indicate improved water retention and physiological resilience, which are key adaptive traits in drought-tolerant plants [46,47].

A major mechanism underlying *IbHK1a*-mediated stress tolerance appears to be the regulation of ROS homeostasis. Abiotic stresses often lead to overproduction of ROS, causing oxidative damage to proteins, lipids, and nucleic acids [48,49]. In this study, histochemical staining revealed significantly lower ROS accumulation in transgenic plants under stress conditions (Figure 5), indicating enhanced detoxification capacity. This was further supported by reduced H_2_O_2_ and MDA levels, suggesting decreased oxidative damage and lipid peroxidation [50]. The enhanced ROS scavenging in *IbHK1a*-overexpressing plants is closely associated with activation of antioxidant enzymes, including SOD, POD, and CAT (Figure 6). These enzymes form the primary defense system against oxidative stress by converting ROS into less harmful molecules [51,52]. The upregulation of antioxidant enzyme activities observed in this study is consistent with previous reports demonstrating that enhanced antioxidant capacity is a key determinant of plant stress tolerance [53,54]. Therefore, *IbHK1a* likely acts upstream of ROS detoxification pathways, linking stress perception to activation of antioxidant defense mechanisms.

The functional role of *IbHK1a* was further validated in sweet potato using a hairy root transformation system. Overexpression lines exhibited improved growth performance and reduced stress-induced damage, whereas RNAi lines showed increased sensitivity under both drought and salt stress conditions (Figure 8). This bidirectional evidence provides strong support for the positive regulatory role of *IbHK1a* in stress tolerance. Similar approaches have been widely used to validate gene function in crop species, demonstrating the reliability of hairy root systems in functional genomics studies [55,56,57,58,59].

Consistent with the *Arabidopsis* results, antioxidant enzyme activities were significantly higher in overexpression lines and lower in RNAi lines, accompanied by corresponding changes in ROS accumulation (Figure 9). This indicates that the mechanism of *IbHK1a*-mediated stress tolerance is conserved between *Arabidopsis* and sweet potato. Conservation of stress-response mechanisms across species is a common feature of key regulatory genes, particularly those involved in core signaling pathways [60].

Functional complementation analysis further demonstrated that *IbHK1a* partially rescues the stress-sensitive phenotype of the *AHK1* mutant (Figure 10A–F), indicating functional conservation with *Arabidopsis AHK1.* However, the incomplete restoration of the mutant phenotype to WT levels suggests that, although *IbHK1a* retains conserved functions, it may not be completely functionally interchangeable with *AtAHK1* in the *Arabidopsis* background. This partial complementation may reflect differences in protein function, regulatory context, or interactions with species-specific signaling components. The findings are consistent with previous research showing that homologous HK genes often retain conserved functions across plant species [61,62]. Furthermore, protein interaction analysis revealed that *IbHK1a* interacts with histidine phosphotransfer proteins (AHP1–AHP5) (Figure 10G), suggesting its involvement in canonical TCS phosphorelay signaling. However, these interactions were demonstrated using a yeast two-hybrid assay and should be further validated using in vivo approaches to confirm their physiological relevance in planta. AHP proteins act as central mediators, transferring phosphate groups from HKs to downstream response regulators, thereby regulating gene expression in response to environmental stimuli [63].

Taken together, these results support a model in which *IbHK1a* acts as a plasma membrane-localized component involved in drought and salt stress responses. The plasma membrane localization of *IbHK1a*, together with its functional conservation with *AtAHK1* and interaction with AHP proteins, is consistent with a model in which *IbHK1a* may participate in stress signal perception and transduction through TCS-mediated phosphorelay pathways. This signaling cascade ultimately activates antioxidant defense systems, enhances ROS scavenging, and improves plant tolerance to abiotic stress. The identification and functional characterization of *IbHK1a* expands our understanding of histidine kinase-mediated stress responses in sweet potato and provides a potential genetic resource for improving stress-resilient crops.

## 4. Materials and Methods

### 4.1. Experimental Materials and Planting Conditions

*Arabidopsis* wild type (Col-0) was bred and preserved in our laboratory, and the T-DNA insertion mutant *ahk1* (SALK_000977) was purchased from the *Arabidopsis* Biological Resource Center (ABRC). *Arabidopsis* seeds were surface sterilized using 20% (*v*/*v*) sodium hypochlorite solution containing 0.1% Tween 20 for 10 min, then washed 3–5 times with sterile double-distilled water and cultured on MS medium. Two-week-old seedlings were transferred to a tray with a culture medium (peat:vermiculite:perlite 3:2:1) for cultivation, maintaining 16 h of light/8 h of darkness at 22 °C. *Nicotiana benthamiana* plants were grown under the same conditions as *Arabidopsis*.

### 4.2. Vector Construction

Construction of *IbHK1a* Gene Promoter Expression Vector: The 2044 bp fragment upstream of the start codon (ATG) was selected as the promoter sequence, and primers were designed (upstream primer *IbHK1apro-KpnI*-F, downstream primer *IbHK1a*pro-*BamHI*-R) (primer sequences are shown in Table S1). Using “Taizhong 6” sweet potato genomic DNA as a template, the corresponding promoter fragment was amplified using KOD high-fidelity enzyme, then cloned into the modified H2B-eGFP-GUS-NOST-pCAMBIA1300-Hyg intermediate expression vector through double digestion with *Kpn*I and *Bam*HI, constructing the *IbHK1apro::*H2B-eGFP-GUS-NOST-pCAMBIA1300-Hyg expression vector.

#### 4.2.1. Construction of 35Spro::*IbHK1a* Overexpression Vector 

The cDNA of sweetpotato ‘Taizhong No.6’ was used as the template, and the amplified target genes were inserted into the pCAMBIA1301 vector.

#### 4.2.2. Construction of ZmUbipro::*IbHK1a* Overexpression Vectors

The cDNA of sweetpotato ‘Taizhong No.6’ was used as the template, and the amplified target genes were cloned into the pTNRT Nmid-EV vector [64].

#### 4.2.3. Construction of pTNRT::*IbHK1a* RNAi Interference Vector

Gene fragments with low sequence homology were selected as interference segments. Specific primer pairs were designed to amplify sense and antisense target fragments separately, which were then cloned into the pEGRNAiP35S vector containing the *cat1* intron.

#### 4.2.4. Construction of 35Spro::*IbHK1a*-GFP Subcellular Localization Vector

The *IbHK1a* CDS fragment was first amplified using primers (upstream primer *IbHK1a* CDS-*Sac*I-F, downstream primer *IbHK1a* CDS-*Kpn*I-R). The fragment was then cloned into the modified 35Spro::eGFP-NOST-pCAMBIA1300-Hyg expression vector via *Sac*I and *Kpn*I double digestion. This resulted in the construction of the 35Spro::*IbHK1a*-eGFP-NOST-pCAMBIA1300-Hyg subcellular localization vector.

#### 4.2.5. Construction of AHK1pro::*IbHK1a* Expression Vector

Based on the published promoter sequence information of *AHK1* [65], the amplified *AHK1* promoter fragment was cloned into the pCAMBIA1301 vector. Subsequently, the target *IbHK1a* fragments were inserted into the pCAMBIA1301 vector carrying the AHK1 promoter.

### 4.3. Genetic Transformation of Arabidopsis and Screening of Transgenic Lines

After sequencing verification, the constructed expression vectors were transformed into *Agrobacterium tumefaciens* strain GV3101 and introduced into *Arabidopsis* WT (Col-0) plants or *AHK1* mutants using the floral-dip method [66,67]. T_1_ seeds were screened on MS medium containing 25 mg·L^−1^ hygromycin B or by glyphosate treatment, depending on the selection marker. Positive transgenic plants were identified by PCR using specific primer pairs for promoter and overexpression constructs. Resistant T_1_ seedlings were transferred to soil (peat moss: vermiculite: perlite, 3:2:1) to obtain T_2_ seeds. T_2_ lines showing a 3:1 segregation ratio were selected, and homozygous T_3_ lines without segregation were used for further experiments.

### 4.4. Genetic Transformation of Sweet Potato Hairy Roots and Screening of Transgenic Lines

The *pTNRT::IbHK1a* overexpression vector*, pTNRT::IbHK1a RNAi* construct, and empty pTNRT vector were introduced into *Agrobacterium rhizogenes* strain K599 by chemical transformation. Positive colonies were selected on LB medium containing 50 mg·L^−1^ kanamycin and 50 mg·L^−1^ streptomycin and cultured at 28 °C for 48 h. Uniform stem segments from “Taizhong 6” sweet potato seedlings were inoculated by injecting A. rhizogenes into internodal regions [64]. The treated explants were planted in a substrate (peat moss: vermiculite: perlite, 3:2:1) and maintained under greenhouse conditions with adequate moisture. After 30 days, transgenic hairy roots were identified by red fluorescence using a LUYOR-3430GR excitation light. Fluorescent roots were further confirmed by PCR using construct-specific primers, and *IbHK1a* expression was analyzed by qRT-PCR. Non-fluorescent roots were periodically removed, and confirmed transgenic hairy roots were selected for subsequent analyses.

### 4.5. GUS Staining Observation

GUS staining of selected *Arabidopsis* transgenic lines was performed as described by [68], with minor modifications. Plant tissues were immersed in GUS staining solution containing X-Gluc and subjected to vacuum infiltration for 30 min, followed by incubation at 37 °C overnight. After staining, chlorophyll was removed using 70% ethanol. GUS signals were observed and photographed using a stereo microscope (SteREO Lumar V12, Carl Zeiss Microscopy GmbH, Jena, Germany) or a microscope (Axioskop 2 plus, Carl Zeiss Microscopy GmbH, Jena, Germany).

### 4.6. Subcellular Localization Observation

The plasmids 35Spro::*IbHK1a*-GFP and the plasma membrane marker (35Spro::PIP2-mCherry) were separately transformed into *Agrobacterium tumefaciens* strain GV3101. Single colonies were picked and cultured overnight at 28 °C with shaking. The bacterial cultures were centrifuged at 4000 rpm for 5 min. Cells were resuspended in infiltration buffer (containing 10 mM MgCl_2_, 50 mM MES (pH 5.6), and 100 μM acetosyringone), and the OD value was adjusted to 1–1.5. Equal amounts of *Agrobacterium* carrying 35Spro::*IbHK1a*-GFP and the plasma membrane marker were mixed and co-infiltrated into the abaxial surface of 30-day-old *Nicotiana benthamiana* leaves. The plants were incubated in darkness at 25 °C for 48 h. Fluorescence signals were observed and photographed using an LSM 710 laser scanning confocal microscope (Carl Zeiss Microscopy GmbH, Jena, Germany).

### 4.7. Germination Rate Statistics of Arabidopsis

The Col-0 WT and T3 generation *Arabidopsis* overexpression lines were surface sterilized, and then the seeds were uniformly sown onto MS solid medium plates containing 0, 100 mM Mannitol, and 100 mM NaCl, with no fewer than 100 seeds per plate, and repeated three times. The plates were first placed in a 4 °C refrigerator for 2 days for vernalization, then transferred to a plant growth chamber with light. The number of seeds that had germinated was counted every 24 h, with germination defined as the rupture of the seed coat. A total of 7 days was recorded, and representative images were captured.

### 4.8. Main Root Length Measurement of Arabidopsis Seedlings

After 3–4 days of vertical cultivation on MS solid medium, seedlings with consistent growth and root length were selected and transferred to MS solid medium plates containing 0, 200 mM Mannitol, and 150 mM NaCl, with 5 seedlings per line, repeated three times. The seedlings were further cultivated for 10 days, after which the root growth was observed, and the main root length of the seedlings was measured using calipers.

### 4.9. Analysis of Chlorosis Rate in Arabidopsis Seedlings

The seed sterilization, sowing, and cultivation methods were the same as described above. After 7 days of vertical cultivation on MS solid medium, seedlings with consistent growth and root length were selected and transferred to MS solid medium plates containing 250 mM NaCl. After 5 days of cultivation, the chlorosis rate of the seedlings was observed and photographed. Chlorosis was defined as when both the cotyledons and true leaves turned completely white.

### 4.10. Measurement of Water Loss Rate

Healthy 4-week-old *Arabidopsis* plants were selected, and six rosette leaves from the same position were excised. Fresh weight was recorded immediately, and leaves were placed in Petri dishes and weighed at 30 min intervals. Each genotype was analyzed with three biological replicates.

### 4.11. Drought and Salt Stress Treatments for Arabidopsis Seedlings

Uniform *Arabidopsis* seedlings grown on MS medium were transplanted into pots containing peat: vermiculite: perlite (3:2:1) and maintained under well-watered conditions for two weeks. For drought treatment, watering was withheld, while control plants continued to receive regular watering [69]. For salt treatment, plants were irrigated every 3 days with Hoagland’s nutrient solution supplemented with 300 mM NaCl, whereas control plants received standard Hoagland’s solution. Plant growth and phenotypic changes were monitored throughout the treatments. After approximately 4 weeks, representative images were captured when clear differences between overexpression lines and WT (WT) plants became evident. For drought-treated plants, rewatering was performed, and survival rates were recorded after 3 days.

### 4.12. Drought and Salt Stress Treatments for Sweet Potato Seedlings

Transgenic sweet potato hairy root lines (overexpression, RNAi, and empty vector) were generated as described in Section 2.4. Uniform and healthy plants were selected and grown in substrate (peat moss: vermiculite: perlite, 3:2:1) under greenhouse conditions. For drought treatment, plants were irrigated with Hoagland’s solution supplemented with 20% PEG6000, while control plants received standard Hoagland’s solution. For salt treatment, a separate set of plants was irrigated with Hoagland’s solution containing 300 mM NaCl, with corresponding controls receiving standard Hoagland’s solution. Both treatments were applied every 3 days, and plant growth was monitored regularly. After approximately 4 weeks, when clear phenotypic differences were observed, representative images were captured. Roots were then washed to remove adhering soil, and fresh weight was measured to evaluate stress responses.

### 4.13. Assessment of Stress-Related Physiological Parameters

Rosette leaves from *Arabidopsis* plants subjected to drought, salt, and control conditions were collected for analysis. Histochemical detection of ROS was performed using DAB and NBT staining kits Beijing Solarbio Science & Technology Co., Ltd., Beijing, China, (Solarbio). After staining, samples were cleared with 75% ethanol and observed under a microscope [70]. For biochemical analyses, MDA content was determined using the Solarbio assay kit (BC0025). H_2_O_2_ levels were measured using kit (BC35950). The activities of antioxidant enzymes, including POD (BC0095), SOD (BC01700, and CAT (BC0205), and ROS were quantified following the manufacturer’s instructions. Chlorophyll content was determined according to the method described by [45]. All measurements were conducted with three biological replicates. Similar physiological assays were carried out on sweet potato roots following stress treatments.

### 4.14. Screening of AHK1 Mutant and Complementation Line Identification

Homozygous *AHK1* mutants were identified using a three-primer PCR strategy with primer combinations LBb1.3/AHK1-RP and AHK1-LP/AHK1-RP. After confirmation, the *AHK1pro::IbHK1a* expression construct was introduced into the ahk1 mutant background via *Agrobacterium*-mediated floral dip transformation, and positive transgenic lines were selected. For T_3_ generation lines, the primary root length of WT, *ahk1* mutants, and complementation lines was measured.

### 4.15. Total RNA Extraction, cDNA Synthesis, and qRT-PCR Methods

Samples were collected from Arabidopsis rosette leaves and sweet potato hairy roots. Total RNA was extracted according to the manufacturer’s protocol, and RNA concentration and purity were assessed spectrophotometrically based on A260/A280 and A260/A230 ratios. The extracted RNA was subsequently used for cDNA synthesis according to the manufacturer’s instructions. qRT-PCR reactions were performed in a total volume of 20 μL containing 10 μL of 2× SYBR Master Mix, 0.4 μL each of forward and reverse primers (10 μM), 2 μL of 10-fold diluted cDNA template, and 7.2 μL of ddH_2_O. The thermal cycling conditions were 95 °C for 2 min, followed by 40 cycles of 95 °C for 15 s, 58 °C for 30 s, and 72 °C for 30 s. Melting-curve analysis was performed to assess amplification specificity. The qRT-PCR assays were further evaluated for amplification efficiency, standard-curve linearity (slope and R^2^), and amplification specificity. Detailed RNA quality measurements and qRT-PCR assay-validation information are provided in Table S2, in accordance with the applicable MIQE recommendations [71]. The reference genes used for normalization were *AtActin7* (AT5G09810) for *Arabidopsis* and *IbActin* (GenBank accession no. EU250003.1) for sweet potato [72]. All PCR and qRT-PCR primers are listed in Table S1.

### 4.16. Yeast Two-Hybrid Assay

Primers were designed to amplify the CDS sequences of AHP1–AHP5 using KOD high-fidelity DNA polymerase. The *pGBKT7* (BD) vector was digested with *EcoRI* and *BamHI*, purified, and recombined with the amplified fragments to generate AHP1–AHP5-pGBKT7 bait constructs. After confirming the absence of self-activation, the bait constructs were co-transformed with the prey vector (*IbHK1a-pGADT7*) into the competent cell strain AH109 for interaction analysis.

## 5. Conclusions

In summary, this study provides evidence that the sweet potato HK gene *IbHK1a* functions as a positive regulator of drought and salt stress tolerance. *IbHK1a* is predominantly expressed in roots and localized to the plasma membrane, consistent with a role in environmental signal perception. Functional analyses in both *Arabidopsis* and sweet potato confirm that *IbHK1a* enhances stress tolerance by promoting root growth, improving physiological performance, and reducing oxidative damage. This enhanced tolerance is closely associated with increased antioxidant enzyme activities and improved ROS-scavenging capacity. Furthermore, the interaction of *IbHK1a* with histidine phosphotransfer proteins and its ability to partially complement the *AHK1* mutant indicate that it operates through a conserved two-component signaling pathway. Collectively, these findings provide new insights into the molecular mechanisms of abiotic stress tolerance in sweet potato and identify *IbHK1a* as a promising candidate gene for developing stress-resilient crop varieties.

## Data Availability

Data presented in this study are available upon request from the corresponding author.

## Supplementary Materials

The following supporting information can be downloaded at: https://www.mdpi.com/article/10.3390/plants15162507/s1, Figure S1: Relative expressions of *IbHK1a* in various transgenic *Arabidopsis thaliana* lines. *IbActin* was used as the internal control; the line with the lowest expression level was considered as ‘1’ with three biological replicates (*n* = 3). Figure S2: Screening and identification of positive transgenic sweetpotato plants. Transgenic hair roots with RFP fluorescence signals 60 days after stem injection of A. rhizogenes bacteria in *IbHK1a*-OE (A), *IbHK1a*-RNAi (B), and vector control (C) sweetpotato plants. (D) Relative expressions of *IbHK1a* in transgenic hair roots. Each treatment has three biological replicates (*n* = 3). ** Indicates *p* < 0.01 and * Indicates *p* < 0.05 according to Student’s *t*-test. Table S1: Primers used in this srudy. Table S2: Detailed RNA, and DNA quality measurements and qRT-PCR assay-validation information.

## Abbreviations

AHK, Arabidopsis Histidine Kinase; AHP, Arabidopsis Histidine Phosphotransfer Protein; CAT, catalase; cDNA, complementary DNA; CDS, coding sequence; DAB, 3,3′-diaminobenzidine; DNA, deoxyribonucleic acid; eGFP, enhanced green fluorescent protein; GFP, green fluorescent protein; GUS, β-glucuronidase; HK, histidine kinase; MDA, malondialdehyde; MS, Murashige and Skoog; NBT, nitroblue tetrazolium; OE, overexpression; PCR, polymerase chain reaction; PEG, polyethylene glycol; POD, peroxidase; qRT-PCR, quantitative reverse transcription polymerase chain reaction; RNA, ribonucleic acid; RNAi, RNA interference; ROS, reactive oxygen species; SOD, superoxide dismutase; TCS, two-component system; VC, vector control; WT, wild type; Y2H, yeast two-hybrid.
