# Supplementary material for: Functional Characterization of IbHK1a Reveals Its Role in Enhancing Drought and Salt Tolerance Through Reactive Oxygen Species Regulation and Two-Component System Signaling in Sweet Potato (Ipomoea batatas L.)
Source: Plants (Basel). 2026 Aug 19;15(16):2507. doi: 10.3390/plants15162507 (PMC13516323; doi:10.3390/plants15162507)
Supplement: Supplementary file 1 [file plants-15-02507-s001.zip › Supplementary Figures.pdf]

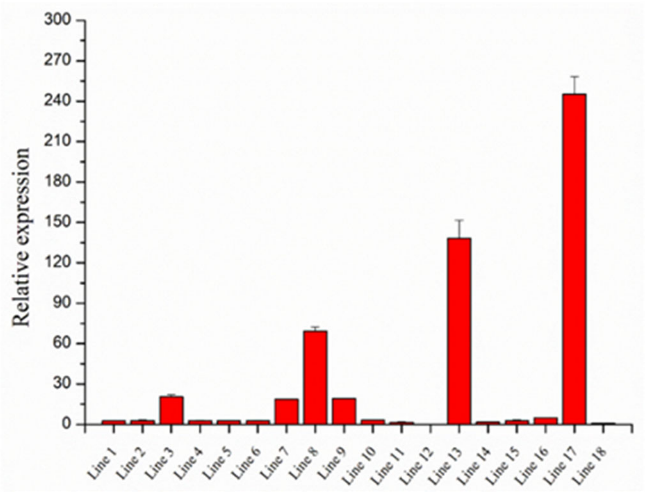

Figure S1. Relative expressions of *IbHK1a* in various transgenic *Arabidopsis thaliana* lines. *IbActin* was used as the internal control; the line with the lowest expression level was considered as '1' with three biological replicates(n=3).

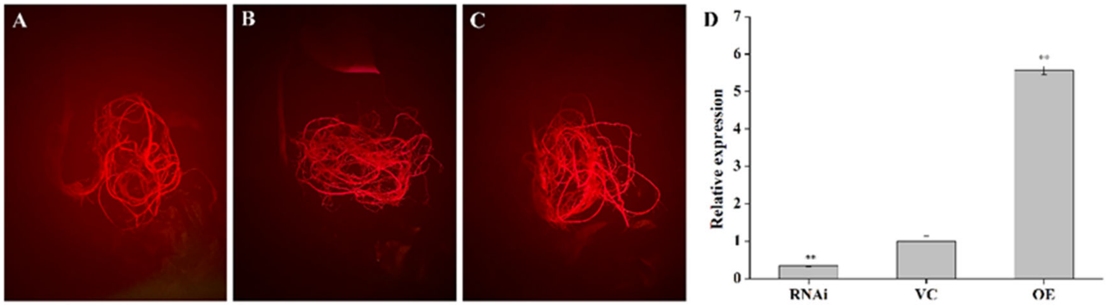

Figure S2. Screening and identification of positive transgenic sweetpotato plants. Transgenic hair roots with RFP fluorescence signals 60 days after stem injection of A. rhizogenes bacteria in *IbHK1a*-OE (A), *IbHK1a*-RNAi (B), and vector control (C) sweetpotato plants. (D) Relative expressions of *IbHK1a* in transgenic hair roots. Each treatment has three biological replicates (n=3). **Indicates $p < 0.01$ and *Indicates $p < 0.05$ according to Student's t-test.
