# Supplementary material for: Functional Characterization of IbHK1a Reveals Its Role in Enhancing Drought and Salt Tolerance Through Reactive Oxygen Species Regulation and Two-Component System Signaling in Sweet Potato (Ipomoea batatas L.)
Source: Plants (Basel). 2026 Aug 19;15(16):2507. doi: 10.3390/plants15162507 (PMC13516323; doi:10.3390/plants15162507)
Supplement: Supplementary file 1 [file plants-15-02507-s001.zip › Supplementary Table S1.pdf]

Table S1. Primers used in this srudy.

Primer name	Primer sequence（5’-3’）	Usage
IbHK1a pro-*Kpn*I-F	GGGGTACCCCAATAAACCATTCACAGTGCTCTT	*IbHK1a* promoter cloning
IbHK1a pro-*BamH*I-R	CGGGATCCTCCTCATATTATTCCCAACAGTAAA	
IbHK1a pro-Seq-R	TTAAGTAGCGAGGAAAATCCACAAT	Promoter expression strain identification
1300-F	CTGGAAAGCGGCAGTGAG	
IbHK1a CDS-*SacI*-F	CGAGCTCATGGCTGTTGGATCCAATACAAGTC	Construction of overexpression vectors and subcellular localization vectors
IbHK1a CDS-*Kpn*I-R	GGGGTACCTCAGTTTTTCTCTTTGTTAATGAA	
IbHK1a CDS-Seq-F	TAAGACAGCGAACGAACAAAAATCC	Identification of Arabidopsis overexpression strains
NOST-HindIII-R	CCCAAGCTTATCGAATTCGATCTAGTAACATAGA	
IbHK1a CDS(TNRT)-F1	GTTCTTCACTGTTGATACAcgcgtATGGCTGTTGGATCCAATACAAGTC	Construction of Sweet Potato Overexpression Vectors
IbHK1a CDS(TNRT)-R1	AGTTGTTGATTCAGAATTGTCGACTCAGTTTTTCTCTTTGTTAATGAA	
IbHK1a CDS-Seq-F	TAAGACAGCGAACGAACAAAAATCC	Identification of sweet potato overexpression lines
HSPT-R	CAAGCCAAGAAAAAAACACAAACT	
NBU4-Seq-F	ATCTTGTTCATTGTTTTGTATTTGC	Identification of Sweet Potato Empty-Vector Transgenic Lines
IbHK1a-T1-F	ActagggtctcGcacc CTGACAAGTCAGAAACTGGGAATGTTAGACC	RNAi vector construction
IbHK1a-T1-R	ActagggtctcTGCAG TGACACTGACAGTGCTTAATTCTACACGACG	
IbHK1a-T2-F	ActagggtctcGGCTT TGACACTGACAGTGCTTAATTCTACACGACG	
IbHK1a-T2-R	ActagggtctcTACCG CTGACAAGTCAGAAACTGGGAATGTTAGACC	
35S-F	CACGGGGGACTCTTGCCACC	Identification of sweet potato RNAi strains
NOST-R	ATCATCGCAAGACCGGCAAC	
AHK1pro-HR-F	ATGATTACGAATTCGAGCTCCTTAAGAG	AHK1 promoter

	CCTATTATGTCTCGCGT	cloning
AHK1pro-HR-R	CTAGAGGATCCCCGGGTACCCTTGTTTCAGATGATCCCAAATCAT	
IbHK1a CDS-rescue-F	TCATCTGAAACAAGGGTACCATGGCTGTTGGATCCAATACAAGTC	Complementation vector construction
IbHK1a CDS-rescue-R	AGGTCGACTCTAGAGGATCCTCAGTTTTTTCTCTTTGTTAATGAA	
ahk1-LP	ATCTCTGCTCTCCTTATCGCC	*Ahk1* mutant identification.
ahk1-RP	GAAGCATCAGACTGGAACGAG	
LBb1.3	ATTTTGCCGATTTCGGAAC	
ARR5-qRT-F	TTGATCGGAAGTTCATCG	*qRT-PCR* Expression Analysis
ARR5-qRT-R	TCATCCCAGGCATAGAGTA	
ARR6-qRT-F	TTTTGCCTCGTATTGATAGATG	
ARR6-qRT-R	CGAGTGAACAGGGTAGACATT	
AtSOD-qRT-F	ATGAGAAGTTCTATGAAGAG	
AtSOD-qRT-R	GTCTTTATGTAATCTGGT	
AtPOD-qRT-F	TCCGGGAGCACACCATTGG	
AtPOD-qRT-R	TGGTCGGAATTCAACAG	
AtCAT-qRT-F	GCAACTACCCCCGAGTGGAAA	
AtCAT-qRT-R	TGTTCAGAACCAAGCGACCA	
AtGPX-qRT-F	ATGGCGACGAAGGAACCAG	
AtGPX-qRT-R	ATCGCCGAAGATTCCCCATTT	
AHP1-pGBKT7-F	TGGCCATGGAGGCCGAATTCATGGATTTGGTTCAGAAGCAGAAG	Construction of the Bait Expression Vector
AHP1-pGBKT7-R	CGCTGCAGGTCGACGGATCCTCAAAATCCGAGTTCGACGGCCGG	
AHP2-pGBKT7-F	TGGCCATGGAGGCCGAATTCATGGACGCTCTCATTGCTCAGCTT	
AHP2-pGBKT7-R	CGCTGCAGGTCGACGGATCCTTAGTTAATATCCACTTGAGGAAC	
AHP3-pGBKT7-F	TGGCCATGGAGGCCGAATTCATGGACACACTCATTGCTCAGTTA	
AHP3-pGBKT7-R	CGCTGCAGGTCGACGGATCCTTATATATCCACTTGAGGGATTCT	
AHP4-pGBKT7-F	TGGCCATGGAGGCCGAATTCATGCAGAGGCAAGTGGCACTCATC	
AHP4-pGBKT7-R	CGCTGCAGGTCGACGGATCCTTACTTGGGCCTACGTGCTGTCTC	
AHP5-pGBKT7-F	TGGCCATGGAGGCCGAATTCATGAACACCATCGTCGTTGCTCAG	
AHP5-pGBKT7-R	CGCTGCAGGTCGACGGATCCCTAATTTATATCCACTTGAGGAAT	
